# Efficacy and Safety of 25% Trichloroacetic Acid Peel Versus 30% Salicylic Acid Peel in Mild-to-Moderate Acne Vulgaris: A Comparative Study

**DOI:** 10.5826/dpc.1103a63

**Published:** 2021-05-20

**Authors:** Surabhi Dayal, Satbir Singh, Priyadarshini Sahu

**Affiliations:** 1 Department of Dermatology, Venereology and Leprology, Pt B D Sharma University of Health Sciences, Rohtak, Haryana, India

**Keywords:** acne vulgaris, trichloroacetic acid, salicylic acid, Michaelsson acne score

## Abstract

**Background:**

Both salicylic acid (SA) and trichloroacetic acid (TCA) have proven efficacy with good safety profiles in the treatment of acne vulgaris.

**Objectives:**

This study compared the clinical efficacy and safety of 25% TCA and 30% SA peels in the treatment of mild and moderate acne vulgaris.

**Methods:**

Patients with mild or moderate acne vulgaris were randomized into 2 groups of 25 persons each, and treated with either the TCA peel or the SA peel at 2-week intervals for 12 weeks. Evaluation of active acne was done by individual lesion counts (comedones, papules and pustules) and calculation of the Michaelsson acne score (MAS).

**Results:**

Both peels led to significant decrease in individual lesion counts and MAS compared to baseline values, without significant differences between the treatment groups. Thus, the peels had equivalent efficacy against acne vulgaris. The TCA peel was better in treating non-inflammatory lesions, while the SA peel was better for inflammatory lesions, but the differences were not significant. No serious adverse effects were recorded, but more patients in the TCA peel group experienced burning and stinging sensations.

**Conclusion:**

The efficacy of 25% TCA is comparable to that of 30% SA in mild-to-moderate acne vulgaris, but safety and tolerability were better with the SA peel than TCA peel.

## Introduction

Acne vulgaris is an inflammatory disorder of the pilosebaceous unit, characterized by noninflammatory lesions (ie, comedones) and inflammatory lesions such as papules, pustules, nodules, cysts and abscesses [1]. A variety of therapeutic modalities are available, including systemic, topical and physical therapies. Topical retinoids, benzoyl peroxide and antibiotics have been the cornerstone of topical treatment of acne [2]. Combination formulations of these topical agents are also commonly prescribed for acne. Recently, topical dapsone, azelaic acid 5%, topical delta-aminolevulinic acid and α-hydroxy acids have been used to treat acne vulgaris [3,4].

Acne vulgaris may be associated with residual pigmentation and scar formation, leading to anxiety, stress and depression. There are various treatment modalities available for acne scars, including chemical peeling, chemical reconstruction using TCA CROSS, dermabrasion, laser treatments, punch techniques, subcision and dermal fillers [5]. Different types of ablative and non-ablative lasers can be used for scar treatment. Ablative lasers include the carbon dioxide laser and erbium-YAG laser [6]. Non-ablative lasers stimulate dermal fibroblasts to produce new collagen. Nd-YAG, diode and recently a new 675-nm Red Touch laser are various types of non-ablative lasers used to treat acne scars [7].

Chemical peel is a well-documented treatment in the management of acne vulgaris and its sequelae, such as post-inflammatory hyperpigmentation (PIH) and scarring [1,8]. In acne vulgaris, both salicylic acid (SA) and trichloroacetic acid (TCA) have proven efficacy as peeling agents with good safety profiles [9–12]. However, there is paucity of studies comparing the therapeutic effect of TCA peel with the more commonly used SA peel, especially in dark-skinned patients [9,10]. Therefore, the present study was undertaken to compare the efficacy and safety of 25% TCA peel versus 30% SA peel for mild-to-moderate facial acne vulgaris in Indian patients.

## Material and Methods

This was a 12-week, prospective, randomized interindividual study. The study was approved by the ethics committee of Pt. B. D. Sharma, University of Health Sciences, Rohtak (approval letter no. IEC/Th/18/SVD/01). Patients with acne vulgaris presenting to the outpatient clinic of the Department of Dermatology were consecutively included in the study. Written informed consent was obtained from all patients aged ≥ 18 years and guardians of the patients age less than 18 years in the study.

### Patient Selection

Patients with mild or moderate facial acne vulgaris (grade I, comedones, occasional papules; and grade II, comedones, many papules, few pustules), as defined by Vaishampayan et al. [3], were eligible for inclusion in the study. Patients were excluded if they had grade III or IV acne vulgaris (ie, with infiltrates, abscesses and nodulocystic lesions); if they were taking any acne medications or had taken oral or topical medications in the past 4 weeks; if they were pregnant or nursing a baby; if they had known hypersensitivity to the formulations used in the study or a history of photosensitivity, hypertrophic scars, keloidal tendency, active or recurrent herpes simplex infection, or any kind of active dermatosis; and if they had unrealistic expectations. A detailed history was taken to rule out all exclusion criteria. A history of all precipitating or initiating factors was taken.

### Treatment

Included patients were randomized into 2 equal groups using a chit-based lottery method. Patients of group 1 were treated with 25% TCA peel and patients of group 2 were given 30% SA peels, at 2-week intervals for a total of 12 weeks. Clinical evaluation was done every 2 weeks throughout the study period. To detect hypersensitivity to the peeling agents, a test peel was done by applying the treatment to the postauricular area.

Peeling was done according to the standard guidelines for chemical peeling [13]. In the TCA peel group, development of uniform erythema as diffuse redness with light cloudy white frosting was considered the desired endpoint. In the SA peel group, immediate whitening, (ie, pseudofrost) within 30 seconds was the end point. After achieving the endpoint, the peel was removed by rinsing with cold water followed by gentle drying with gauze. Any acute minor side effects related to the therapy were treated with appropriate medication by an investigator.

### Clinical Assessment of Efficacy

Clinical photographs of each patient were taken at 2-week intervals, with front, right and left views of the face. Michaelson acne scores (MAS) [14] were calculated at baseline and at each visit. Acne improvement was graded according to the reduction in mean MAS between baseline and 12 weeks, and evaluated as good when greater than 50%, fair when 21%–50%, and poor when less than 20%.

### Statistical Analysis

The Statistical Package for Social Sciences (SPSS) for Microsoft Windows 20th version was used for statistical analysis. For the comparison of nominal or continuous data such as age distribution, duration of disease, individual lesion count and MAS within the group and between the groups, paired and unpaired Student’s t tests were used, respectively. Categorical data, ie, sex of the patients and improvement in acne in each group, were compared using the chi-squared test. The tests were performed at a 5% level of significance and an association was found to be significant if the P value was <.05.

## Results

**Figure 1:**
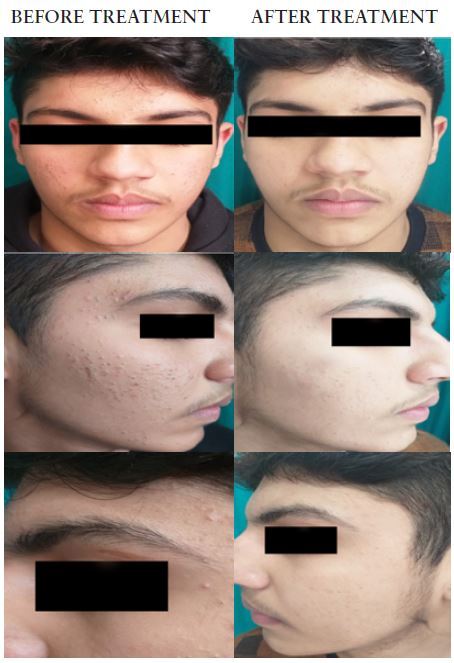
Improvement in lesions of acne vulgaris before and after TCA peel.

**Figure 2:**
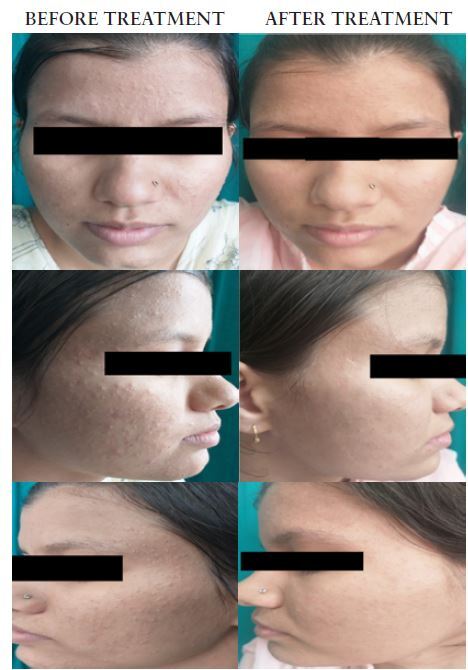
Improvement in lesions of acne vulgaris before and after SA peel.

A total of 50 patients with mild or moderate acne vulgaris were included in the study and randomized to treatment with either a 25% TCA peel or 30% SA peel. The groups were comparable with respect to age distribution, sex, duration of disease and mean MAS at baseline (Table 1). There were no statistically significant differences between the groups with respect to mean comedo, papule and pustule counts before starting the therapy. Before-after clinical photographs for one patient in each group are shown in Figures 1 and 2.

### Evaluation of Clinical Efficacy

**Figure 3:**
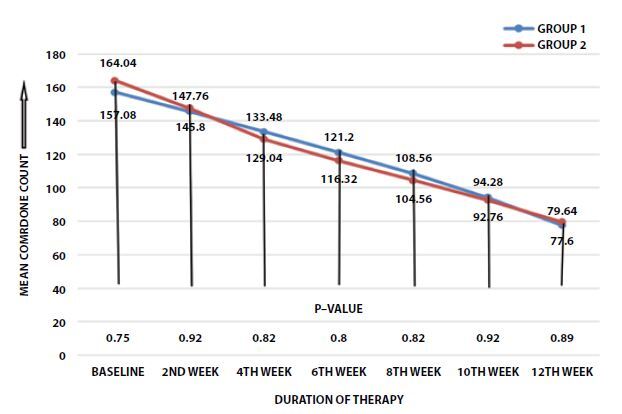
Mean comedo counts in Group 1 (TCA peel) and Group 2 (SA peel) throughout the treatment period.

The mean comedo counts at the end of therapy were significantly lower than the baseline values in both groups (Figure 3). The decrease started after 2 weeks of therapy and remained statistically significant throughout the therapy. However, there was no difference between the two groups in terms of the change in comedo counts at the end of therapy (P = .89). The mean percentage decrease in comedo counts from baseline in group 1 was 53.91% (SD = 9.43%) and in group 2 53.71% (SD = 13.66%), without a significant difference (P = .95).

**Figure 4:**
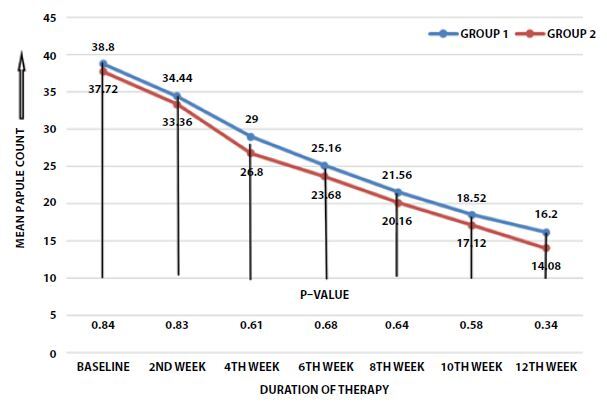
Mean papule counts in Group 1 (TCA peel) and Group 2 (SA peel) throughout the treatment period.

There was significant decrease in mean papule count from the baseline values in both groups at the end of 12 weeks of therapy (Figure 4). The decrease started at 2 weeks and remained significant throughout the therapy. However, the difference between the groups was not significant at the end of therapy (P = .34). The percentage decrease in mean papule count in group 1 was 56.68% (SD = 13.12%) and in group 2 59.93% (SD = 13.94%), without a significant difference (P = .4) after 12 weeks of therapy.

**Figure 5:**
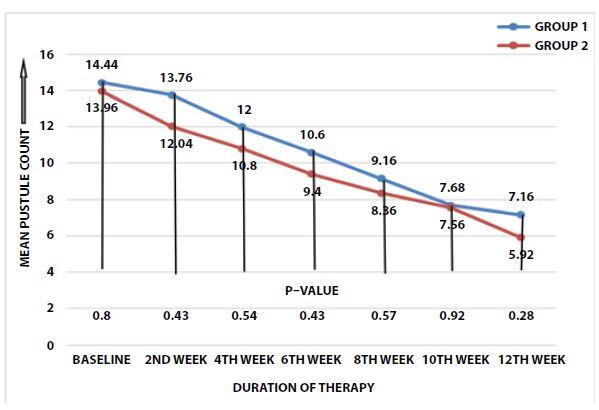
Mean pustule counts in Group 1 (TCA peel) and Group 2 (SA peel) throughout the treatment period.

There was significant decrease in mean pustule count from the baseline values in both groups at the end of 12 weeks of treatment (Figure 5). A significant decrease in mean pustule count from baseline was observed in group 1 at the end of 4 weeks, while the decrease in mean pustule count started earlier, ie, at the end of 2 weeks in group 2. However, the difference between the groups in terms of pustule count was not significant at the end of therapy (P = .28). The percentage decreases in mean pustule count in groups 1 and 2 were 51.98% (SD = 19.32%) and 55.15% (SD = 18.01%), respectively, but the difference was not significant (P = .55).

**Figure 6:**
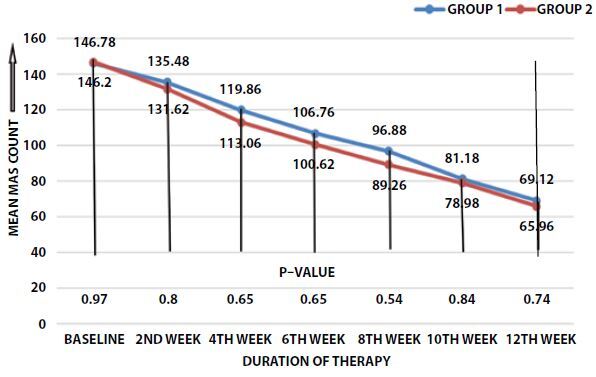
Mean Michaelsson acne scores (MAS) in Group 1 (TCA peel) and Group 2 (SA peel group) throughout the treatment period.

There was significant decrease in mean MAS in both groups from baseline to the end of therapy (Figure 6). The significant decrease in mean MAS was observed at the end of 2 weeks in both groups. However, the difference in mean MAS between the groups was not significant at the end of therapy (P = .74). On comparing the groups in terms of percentage decrease in mean MAS at the end of 12 weeks with respect to baseline, group 2 showed slightly better results with a percentage decrease of 55.97% (SD = 11.32%) as compared to 54.64% (SD = 9.32%) in group 1. However, the difference between the groups was not significant (P = .65).

On analyzing the change in MAS between the start and end of therapy, we found that all patients had good or fair improvement and none had poor improvement. Good improvement (>50% decrease in MAS) was seen in 15 of the 25 patients in group 1 (TCA peel) and in 17 patients of group 2 (SA peel), without a significant difference (P = .54). Fair improvement (20%–50% decrease in MAS) was present in 10 patients in group 1 and 8 patients in group 2 (P = .52).

### Adverse Effects

As far as side effects are concerned, burning and stinging sensations were more common in group 1 (20 of 25 patients) than in group 2 (10 of 25 patients); this difference was statistically significant (P = .004). Post-peel erythema was also more common in group 1 (10 of 25 patients) than in group 2 (4 of 25 patients), but this difference was not significant (P = .05). PIH was observed in 5 patients in group 1 and 2 patients in group 2 (not significant). It resolved on its own in group 2, while in group 1 it resolved after treatment with topical application of mild desonide cream and strict sun protection for 1 week. No patient in the study had blistering, crusting, scaling, hypertrophic scarring or keloid formation.

## Discussion

Chemical peeling is a well-known treatment for acne. Peeling agents that have been used in the treatment of acne vulgaris include alpha hydroxy acids (eg, glycolic acid, lactic acid, mandelic acid), beta hydroxy acids (eg, salicylic acid, lipohydroxy acid), tretinoin peels, TCA peels and Jessner’s solution [1,9–12,15–17]. TCA peel is effective for histologically and clinically improving the skin in a variety of dermatological conditions [18,19]. It has been used to treat acne, either alone or in combination with other drugs. SA peel (20%–30%) is a well-established superficial peeling agent for the treatment of acne vulgaris [3], and its efficacy has been documented by several studies [3, 8–12]. SA is effective against both acne and PIH, which are common in people with skin of dark color. Its whitening effect is an important factor in its choice as a superficial peeling agent for Asian patients with acne vulgaris [20].

We found only 2 studies that compared the efficacy and safety of SA and TCA peels [9,10]. In a recent study by Abdel Hay et al [10], a combination solution of 20% azelaic acid and 20% SA was compared with 25% TCA peel in 34 patients with mild or moderate acne vulgaris. At the end of 8 weeks, significant improvements were seen in both treatment groups. However, the difference between the 2 treatments was not significant. According to the authors, the combination of azelaic acid and SA is recommended in the early stage of the disease, ie, when patients have inflammatory lesions, while TCA is preferred for patients with non-inflammatory lesions. In a comparative, split-face study by Abdel Meguid et al [9], 25% TCA peels and 30% SA peels were compared in 20 patients of Fitzpatrick skin types III to V with facial acne. At the end of the study, total improvement was more frequent with the SA than TCA peel, but the difference was not statistically significant. Total improvement in comedones was more frequent with TCA peeling, while improvement of inflammatory lesions was more frequent on the side treated with the SA peel. However, the results did not reach the level of statistical significance.

Our study enrolled 50 patients with mild or moderate acne vulgaris. Objective evaluation of active acne was done by individual lesion counts (comedones, papules and pustules) and calculation of MAS. In terms of improvement in non-inflammatory lesions (ie, comedones), both peels brought a significant decrease in mean comedo counts from their respective baseline values. The percentage decrease in non-inflammatory lesions was slightly more with 25% TCA peel than 30% SA peel; however, the difference was not significant at the end of therapy. This finding is in agreement with the study by Abdel Meguid et al [10], and may be due to fact that both TCA peel and SA peel have comedolytic action.

The mechanism of action of TCA peel in the treatment of acne vulgaris is due to its ability to diminish corneocyte cohesion and keratinocyte plugging, thus helping in comedolytic action. In addition, application of TCA to the skin causes precipitation of proteins and coagulative necrosis of epidermal cells, leading to removal of damaged skin and its replacement by normal tissue [19]. The effect of SA is mainly due to the lipophilic activity and comedolytic effect. The initial event in comedo formation is excessive keratinization in the mid-portion of the follicular canal; due to its lipophilic nature, SA preferentially acts on the sebaceous unit which is required and important for comedolysis [9]. Since both TCA and SA facilitate comedolysis, the effectiveness of both peels with respect to comedolytic action may be comparable.

In our study, a significant decrease in mean papule count was observed after 2 weeks of therapy, but there was no significant difference between the groups at the end of therapy. The overall percentage decrease in mean papule count was better in group 2 (SA peel group) than in group 1 (TCA peel group), but the difference was not significant. Similarly, a significant decrease in mean pustule count started earlier (ie, at 2 weeks) in the SA peel group than TCA peel group in which it was seen at 4 weeks of therapy. Furthermore, the overall percentage decrease in mean pustule count was better in the SA peel group than the TCA peel group, but the difference was not significant. Thus, there was statistically significant decrease in mean pustule count after completion of therapy in the 2 groups, but the difference was not significant at the end of therapy. The study by Abdel Meguid et al [9] found that 30% SA peels are superior to 25% TCA peels for treating inflammatory lesions in dark-skinned patients. Thus, the results of our study regarding the improvement in inflammatory acne lesions are in agreement with that study. The better effects in terms of improvement of papules and pustules (inflammatory lesions) with the SA peel than TCA peel may be due to the anti-inflammatory action of SA through inhibition of the arachidonic acid cascade [21,22].

On analyzing the improvement in MAS, there was a statistically significant difference from baseline values to the end of therapy in both groups, but the difference between groups was not significant. Thus, our result is in agreement with those of Abdel Meguid et al [9], who also found that the efficacy of 25% TCA peels and 30% SA peels are comparable in the treatment of mild-to-moderate acne vulgaris.

As far as side effects are concerned, patients of both groups tolerated the peels very well. However, the SA peel was found to be safer in terms of side effects like erythema and PIH, and was superior as far as burning and stinging sensations were concerned. No patient experienced any serious adverse effect requiring cessation of therapy. However, as skin of color is more prone to developing PIH, the SA peel seems to be the better choice for dark skin owing to the additional advantage of its whitening effects [15,20]. The anti-inflammatory action of SA further adds to its beneficial effects as compared to TCA peel [21,22]. Furthermore, our patients also demonstrated better tolerability to the SA peel than the TCA peel in the form of less burning and stinging, which enhances the compliance of patients with skin of color.

Our study has strengths related to the relatively larger study group and the use of an objective method to analyze the improvement of acne, ie, calculation of MAS. However, there are also a few limitations to our study. Firstly, follow-up was not done to determine the recurrence rate among the patients. Secondly, we did not evaluate the patients’ satisfaction with the treatments.

## Conclusions

Our study demonstrated that the efficacy of 25% TCA peel is comparable to that of the 30% SA peel in the treatment of mild or moderate facial acne vulgaris in Indian patients. Furthermore, the 30% SA peel is marginally better than the 25% TCA peel for inflammatory lesions, while for non-inflammatory lesions 25% TCA seems better. In terms of safety and tolerability, the 30% SA peel was better than the 25% TCA peel, as a greater number of patients in the 25% TCA peel group developed adverse effects such as burning, stinging, PIH and post-peel erythema than in the 30% SA peel group. Hence, this study infers that although the therapeutic efficacies of the 25% TCA and 30% SA peels are comparable in Indian acne vulgaris patients, the 30% SA peel seems to be the treatment of choice for Indian patients due to its lightening effects and the lesser chance of causing PIH.

## Figures and Tables

**Table 1:** Baseline Characteristics of the 50 Patients with Acne Vulgaris

	TCA peel group (n = 25)	SA peel group (n = 25)	P

Age (years), mean (SD)	17.9 (2.4)	17.8 (1.9)	.95
Sex, n			.56
Male	9	11	
Female	16	14	
Disease duration (months), mean (SD)	18.24 (17.92)	24.24 (21.56)	.075
Comedone count, mean (SD)	157.08 (83.49)	164.04 (70.96)	.75
Papule count, mean (SD)	38.8 (18.06)	37.72 (20.46)	.84
Pustule count, mean (SD)	14.44 (5.8)	13.96 (7.88)	.8
MAS, mean (SD)	146.2 (59.62)	146.78 (51.27)	.97

MAS = Michaelsson acne score; SA = salicylic acid; SD = standard deviation; TCA = trichloroacetic acid.
